# A case report of secondary B-cell acute lymphoblastic leukemia treated with a combination of FLT3 inhibitor and decitabine

**DOI:** 10.3389/fonc.2024.1329279

**Published:** 2024-04-26

**Authors:** Mengci Hu, Wenzhe Li, Pan Li, Jie Tan, Ya Wang

**Affiliations:** ^1^ Department of Hematology, The First Affiliated Hospital of Yangtze University, Jingzhou, Hubei, China; ^2^ Department of Endocrinology, The First Affiliated Hospital of Yangtze University, Jingzhou, Hubei, China; ^3^ Department of Hubei Provincial Clinical Research Center for Personalized Diagnosis and Treatment of Cancer, The First Affiliated Hospital of Yangtze University, Jingzhou, Hubei, China

**Keywords:** diffuse large B-cell lymphoma, acute lymphoblastic leukemia, secondary acute lymphoblastic leukemia, FLT3, myelodysplastic syndrome

## Abstract

Secondary acute lymphoblastic leukemia (s-ALL) refers to acute lymphoblastic leukemia that occurs after a previous malignant tumor, including therapy-related acute lymphoblastic leukemia (t-ALL) and prior malignant tumor acute lymphoblastic leukemia (pm-ALL). We report a case of a 51-year-old female patient who developed acute lymphoblastic leukemia 14 years after being diagnosed with diffuse large B-cell lymphoma (DLBCL). The patient was unresponsive to conventional chemotherapy for acute lymphoblastic leukemia (ALL) and achieved remission with a combination of sorafenib and decitabine based on the molecular biology characteristics of her B-ALL.

## 1 Introduction

The therapy related acute lymphoblastic leukemia (t-ALL) is defined as malignant tumor patients who have been exposed to radiation and/or chemotherapy in the past (1). Due to the rarity of the disease, our understanding of t-ALL is limited, but some current studies suggest that t-ALL is a unique entity with poor genetic characteristics and clinical outcomes (2, 3). There is currently no standard treatment for t-ALL, and the response rate to traditional therapies is low with poor survival outcomes. The application of molecular research and next-generation sequencing technologies to identify genetic characteristics may be helpful in selecting treatment plans, but long-term survival still depends on allogeneic hematopoietic stem cell transplantation.

## 2 Case presentation

The patient is a 51-year-old female who initially presented with an upper abdominal mass in 2009. A surgical resection of the mass was performed and subsequent pathological examination revealed a diagnosis of diffuse large B-cell lymphoma (DLBCL), stage IA, with an International Prognostic Index (IPI) score of 1 point, placing her in the low-risk group. Following six cycles of cyclophosphamide, pirarubicin, vindesine, and dexamethasone (CHOP) chemotherapy, she achieved complete remission. In June 2013, a relapse of DLBCL in the breast occurred. The patient underwent an additional 6 cycles of CHOP chemotherapy and received intermittent intrathecal injections of methotrexate, cytarabine, and dexamethasone to prevent central nervous system infiltration of lymphoma. Once again, the patient achieved complete remission.

In August 2019, the patient was diagnosed with central nervous system lymphoma. Further diagnostic procedures, including marrow aspiration cytology and flow cytometry, confirmed the diagnosis as DLBCL stage IV, with an IPI scored of 2, placing the patient in the medium-low risk group. The treatment plan involved craniospinal irradiation, which resulted in a significant reduction of intracranial lesions as observed in a follow-up MRI conducted one month later. Subsequently, from January 2020 onwards, the patient underwent 7 cycles of a combination of Rituximab and lenalidomide (R2), followed by maintenance therapy involving lenalidomide.

**Figure 1:**
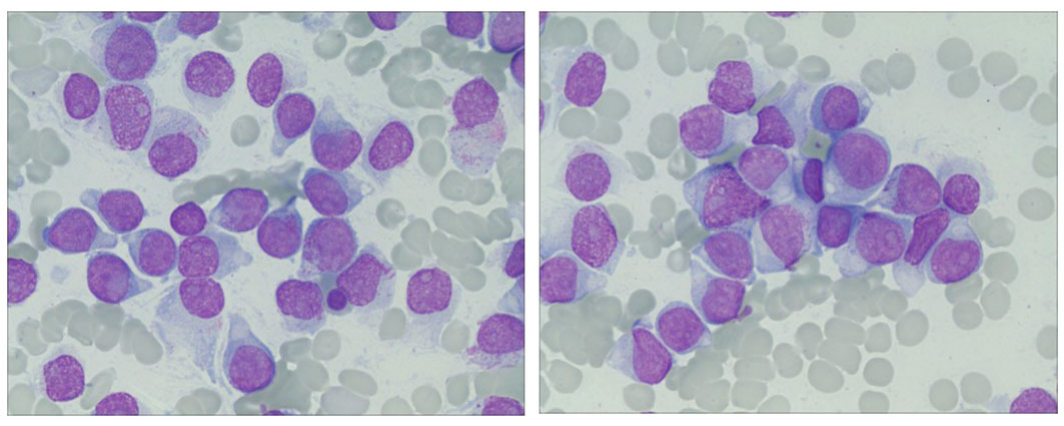
Ring sideroblasts in bone marrow. The bone marrow aspirate from the patient showed abnormal lymphocytes occupying 94% of the nucleated cells.

**Figure 2:**
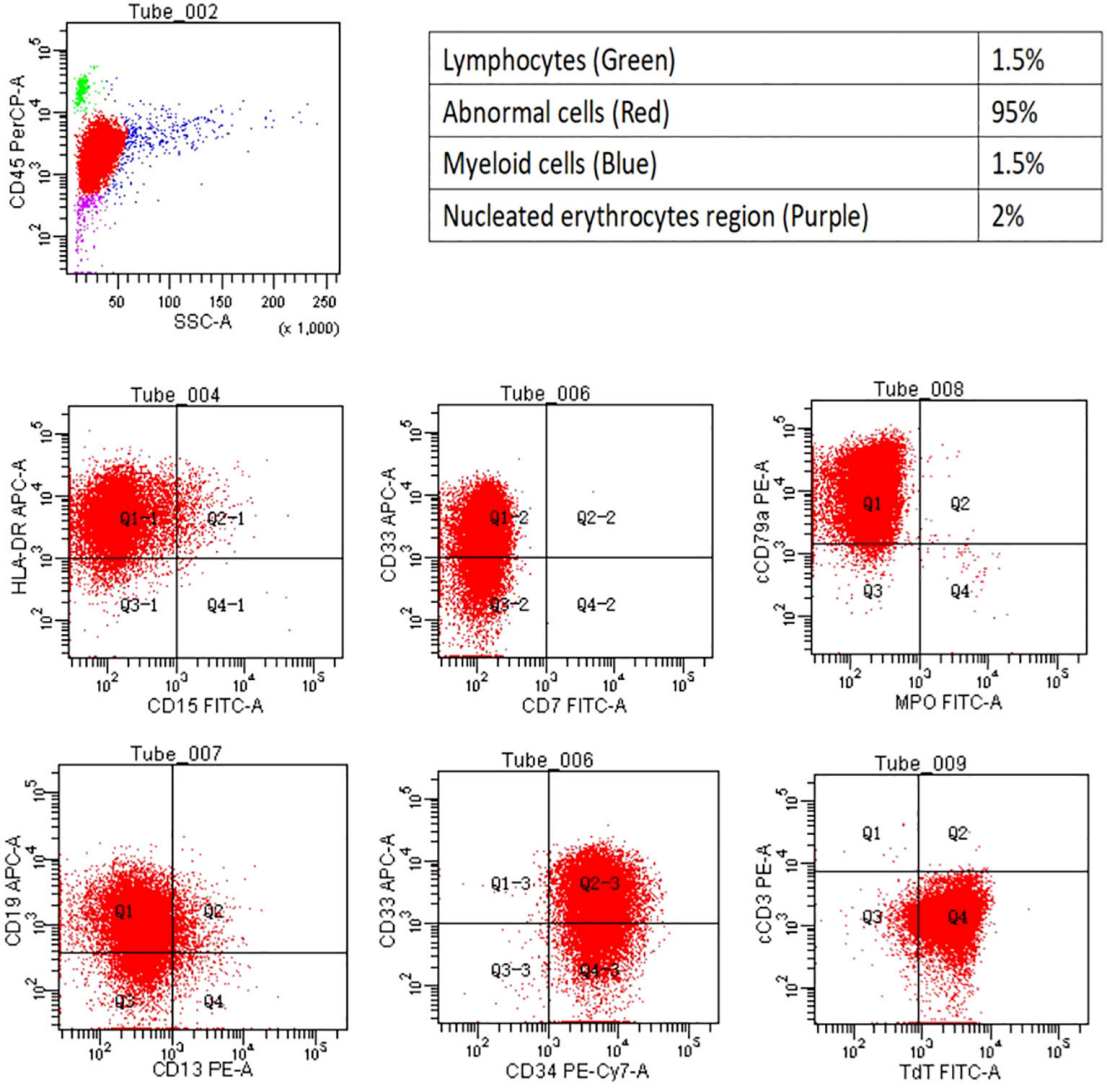
Flow cytometry. The flow cytometry analysis revealed the cell population proportion of nucleated cells and their positivity for HLA- DR, CD19, CD33, CD34, cCD79a, and TDT.

In April 2023, the patient’s blood cell count revealed a total white blood cell count of 21.59x10^9/L, platelet count of 70x10^9/L, hemoglobin of 108.00g/L, lymphocyte percentage of 44.40%, monocyte percentage of 52.30%, and neutrophil count of 0.64x10^9/L. Notably, bone marrow cytology analysis demonstrated that abnormal lymphocytes comprised 94% of nucleated cells, suggesting a new diagnosis of acute lymphoblastic leukemia (**Figure 1**). Flow cytometry analysis also confirmed the presence of abnormal lymphocytes expressing HLA-DR, CD19, CD33, CD34, CD38, CD58, CD123, cCD79a, and TdT (**Figure 2**), indicating a diagnosis of acute B lymphoblastic leukemia (pro-B-ALL possible). BCR::ABL1 p190 was found in the screening of 56 common fusion genes, with a quantification of 0.03%. Chromosomal karyotype analysis showed a normal female karyotype, 46 XX [20]. The Next-generation sequencing (NGS) detected four class I mutations: FLT3 (p.Y572C and p.I836delI variants, with mutation allele frequencies (MAF) of 21.28% and 5.18% respectively), BCORL1 (p.D1495Efs7 and p.R1090X variants, with MAFs of 22.63% and 0.78% respectively), XBP1 (p.R168Sfs196 variant, with a MAF of 44.56%), and DNMT3A (c.1015-3_1015-2delinsAG variant, with a MAF of 42.88%). After being hospitalized, the patient received induction chemotherapy consisting of dexamethasone 10mg + vindesine 4mg, with the addition of imatinib treatment. One week later, a review of bone marrow cytology revealed that primitive and immature lymphocytes still accounted for 70.5% of the total. The treatment plan was adjusted to include a combination of decitabine 20mg/m^2^ on days 1-5, sorafenib 0.4g twice daily, and dasatinib 70mg once daily. One week after completing this chemotherapy regimen, a follow-up bone marrow aspiration cytology showed that the proportion of primitive cells had decreased to 0.5%. Minimal residual disease (MRD) detection indicated approximately 0.4% of abnormal B lymphocytes. In May 2023, the patient’s BCR::ABL1 fusion gene and MRD were negative, at which point we discontinued the use of TKIS. Up to now, the detection of BCR::ABL1 emains negative. During the course of treatment with decitabine and sorafenib, the patient experienced transient leukopenia and localized erythema and edema of the fingers. Currently, the patient has completed 8 cycles of decitabine and sorafenib, and remains in complete remission.

## 3 Discussion

The incidence of t-ALL accounts for 3%-9% of all cases of ALL (4). It is further categorized into alkylating agent/radiotherapy-related leukemia and leukemia that occurs after treatment with DNA topoisomerase II inhibitors (5). Similar to treatment-related acute myeloid leukemia (t-AML) or treatment-related myelodysplastic syndrome (t-MDS), the pathogenesis of t-ALL is also attribute to the genotoxic effects of cytotoxic therapy on hematopoietic stem cells. However, the exact mechanism underlying t-ALL development is not as clear as that of treatment-related myeloid tumors (6). The median latency period for t-ALL diagnosis is approximately 6.8 years. It is commonly associated with advanced age, female gender, a previous history of breast neoplasms, and central nervous system tumors (2, 3, 7). Studies have reported that maintenance therapy involving lenalidomide and thalidomide may contribute to the development of t-ALL (7–9). The mechanism may involve lenalidomide inducing the proteasomal degradation of the transcription factor Ikaros encoded by IKZF1, while mutations or deletions in IKZF1 have been shown to play a driving role in the development of ALL (4). The median time for lenalidomide-induced t-ALL onset is approximately 32.5 months (10).

Compared to newly diagnosed ALL (dn ALL), the occurrence of high-risk cytogenetic and molecular features is higher in t-ALL. These features include BCR::ABL1 positivity, MLL rearrangements, and hyperdiploid (3, 7, 11, 12). Several studies have also indicated that the incidence of BCR::ABL1 positivity is similar between these two types of leukemia (3, 13). The most commonly mutated genes in dn ALL were IKZF1 (37%), CDKN2A (14%), SETD2 (13%), and CDKN2B (11%). On the other hand, TP53 (38%) and RB1 (25%) were the most frequently mutated genes in t-ALL (3). One study has reported that that most t-ALL patients had mutations more commonly found in myeloid malignancies, such as DNMT3A, RUNX1, and ASXL1. Some patients also had ALL-type mutations, like CDKN2A and IKZF1, or mutations in other cancer susceptibility genes, such as BRCA2 (2).

In this case, the patient is a middle-aged woman who was initially diagnosed with DLBCL. The patient had undergone radiation therapy and multiple rounds of chemotherapy, she also had a history of breast and central nervous system lymphoma and she had been exposed to lenalidomide for 27 months, all of which are consistent with the characteristics of t-ALL. Moreover, the second-generation sequencing did not reveal mutations in commonly observed genes in DLBCL, and flow cytometry analysis of lymphocytes revealed an immunophenotype consistent with pro-B-cell ALL. Therefore, it is considered that the patient has developed treatment-related lymphocytic leukemia. Due to the prolonged duration and complex treatment process of this patient, it is difficult to ascertain whether any single drug or radiotherapy-induced secondary lymphocytic leukemia during treatment. In this particular case, four Class I mutations detected (FLT3, DNMT3A, XBP1, BCORL1) are not commonly observed in patients with ALL. The incidence of FLT3 gene mutation in adult and pediatric B-ALL patients is 12.3% and 2%, respectively (14). This mutation is associated with a poor prognosis, however, patients with this mutation have shown potential responses to tyrosine kinase inhibitors (15). Based on this premise, the FLT3 inhibitor sorafenib was chosen for the treatment regimen of the patient. DNMT3A mutations are known to be associated with a poor prognosis in acute myeloid leukemia (AML) and T-lymphoblastic leukemia (T-ALL), but the expression level and prognostic significance of DNMT3A in B-ALL remain unclear (16). DNMT3A is one of the commonly mutated genes in Clonal Hematopoiesis of Indeterminate Potential (CHIP) (17). It may promote irregular cell proliferation, increasing the risk of hematological (17, 18). Previous data from AML indicate that DNMT3A gene mutations increase sensitivity to hypomethylating agents, which provides a rationale for utilizing decitabine in this patient.

Previous reports indicate that patients with t-ALL can achieve a similar remission rate to dn ALL after conventional chemotherapy (1, 7, 11). However, t-ALL patients exhibit a significantly lower overall survival (OS) rate, with median OS ranging from 20 weeks to 13.6 months based on various studies (12). The presence of s-ALL is considered to be an unfavorable independent prognostic factor, potentially due to its adverse cytogenetic and molecular characteristics (12, 19, 20). Other factors contributing to poorer OS in t-ALL patients include advanced age at diagnosis and prior exposure to radiation therapy or chemotherapy, which may potentially induce mutations associated with secondary malignancies (21–24). Currently, there is limited research on post-conventional chemotherapy failure treatment. According to the NCCN guidelines for Acute Lymphoblastic Leukemia, chemotherapy is not the first-line treatment for refractory/relapsed Ph-negative B-ALL. The preferred treatment options recommended by the guidelines include blinatumomab, inotuzumab ozogamicin, tisagenlecleucel, and brexucabtagene autoleucel. Other recommended regimens include inotuzumab ozogamicin + mini-hyper-CVD ± blinatumomab (cyclophosphamide, dexamethasone, vincristine, alternating with methotrexate, cytarabine), clofarabine alone or in combination, MOpAD regimen, fludarabine-based regimens, cytarabine-containing regimens, and alkylator combination regimens, and more (25). Chimeric antigen receptor-engineered T (CAR-T) cell therapy has achieved a complete remission rate of 60%-90% in adult and pediatric relapsed/refractory ALL (26). But these only achieve short-term remission. Allogeneic hematopoietic stem cell transplantation (allo-HSCT) shows promise in improving long-term outcomes for t-ALL patients (1, 13). For those undergoing allo-HSCT, factors such as exposure to certain agents or genetic anomalies do not exhibit an adverse correlation with relapse-free survival (RFS) or OS. This emphasizes the potential of allo-HSCT in mitigating the negative impact associated with these factors (1). For this case, Blinatumomab and CAR-T cell therapy may present as feasible therapeutic strategies. However, she has declined both treatment options. Therefore, in response to the aforementioned gene mutations and BCR::ABL1 fusion gene positivity, second-generation ABL kinase inhibitor dasatinib, FLT3 inhibitor sorafenib, and demethylating agent decitabine were selected. Following one cycle of treatment, the patient achieved complete remission. It is noteworthy that the quantification of this patient’s BCR::ABL1 fusion gene was exceptionally low, which we believe could be attributed to it being a subclone. Following treatment and disease remission, this subclone was eliminated.

## 4 Conclusions

In summary, t-ALL is a relatively rare type of leukemia. At present, there is no standard treatment regimen, and the efficacy of conventional chemotherapy regimens is not satisfactory. Personalized treatment plans based on patient-specific gene mutations and immunological targets have shown some promise in achieving certain levels of effectiveness. However, the remission period tends to be short, and the long-term survival rate remains low. Allo-HSCT may be a viable treatment option. Further research is still required to explore the potential relationship between t-ALL and specific chemotherapy or radiotherapy regimens, as well as the genetic and molecular characteristics of t-ALL, in order to develop more appropriate and effective treatment strategies.

## Data availability statement

The original contributions presented in the study are included in the article/supplementary material. Further inquiries can be directed to the corresponding authors.

## Ethics statement

The studies involving humans were approved by Medical Ethics Committee of the First People’s Hospital of Jingzhou. The studies were conducted in accordance with the local legislation and institutional requirements. The participants provided their written informed consent to participate in this study. Written informed consent was obtained from the individual(s) for the publication of any potentially identifiable images or data included in this article.

## Author contributions

MH: Writing – original draft. WL: Writing – original draft. PL: Writing – original draft. JT: Writing – review & editing. YW: Writing – review & editing.
